# Cell Cycle Regulators and Lineage-Specific Therapeutic Targets for Cushing Disease

**DOI:** 10.3389/fendo.2018.00444

**Published:** 2018-08-10

**Authors:** Takako Araki, Ning-Ai Liu

**Affiliations:** ^1^Division of Diabetes, Endocrinology and Metabolism, Department of Medicine, University of Minnesota, Minneapolis, MN, United States; ^2^Department of Medicine, Pituitary Center, Cedars-Sinai Medical Center, Los Angeles, CA, United States

**Keywords:** pituitary tumor, Cushing disease, cell cycle, cyclin E, E2F1, POMC

## Abstract

Cell cycle proteins are critical to pituitary development, but their contribution to lineage-specific tumorigenesis has not been well-elucidated. Emerging evidence from *in vitro* human tumor analysis and transgenic mouse models indicates that G1/S-related cell cycle proteins, particularly cyclin E, p27, Rb, and E2F1, drive molecular mechanisms that underlie corticotroph-specific differentiation and development of Cushing disease. The aim of this review is to summarize the literature and discuss the complex role of cell cycle regulation in Cushing disease, with a focus on identifying potential targets for therapeutic intervention in patients with these tumors.

## Introduction

Pituitary corticotrophs play critical physiologic roles in hypothalamic–pituitary–adrenal axis functions, including the acute stress response, regulation of body metabolism and energy expenditures, and immune function (1). Patients with Cushing disease caused by a pituitary corticotroph adenoma can manifest obesity, diabetes, susceptibility to infections, psychosis, and coagulopathy, which contributes to increased mortality (2). Surgical tumor resection is the primary therapy for Cushing disease, but persistent/recurrent disease is seen in 12–40% of patients depending on surgical expertise, the definition of remission, and the duration of follow-up (3, 4). Pituitary-directed radiation and bilateral adrenalectomy to inhibit adrenal cortisol production are effective at inducing biochemical control but are infrequently used. Response to radiation therapy is slow and risk of hypopituitarism is high. Following adrenalectomy, lifelong replacement glucocorticoid and mineralocorticoid is needed, and the loss of negative feedback on pituitary adrenocorticotropic hormone (ACTH) can lead to tumor growth (5, 6).

Safe and effective medical management of Cushing disease has also proven challenging. The steroidogenesis inhibitors ketoconazole and metyrapone normalize cortisol in about 50% of cases, but with a risk of inducing adrenal insufficiency or hepatotoxicity (7, 8). The investigational steroidogenesis inhibitor osilodrostat showed improved responses, with up to 80% achieving biochemical remission, but still carries the risk of adrenal insufficiency and hepatotoxicity, and the loss of feedback led to a 4-fold increase in ACTH (9). The glucocorticoid receptor blocker mifepristone, approved for treatment of diabetes due to Cushing's syndrome, improves the systemic effects of excess cortisol on glycemia and weight, but is associated with risk of adrenal insufficiency, hypokalemia, and endometrial thickening, and may induce tumor growth (10). Importantly, none of these agents directly target the tumor.

Currently, two tumor-targeting agents are used in Cushing disease (11). The dopamine agonist cabergoline targets D2 receptors in the tumor and biochemical control is seen in 30–40% of patients after treatment with relatively high doses of 2–3 mg/week for at least 2 years (12, 13). However, cabergoline is not approved for use in this disease. The somatostatin receptor ligand pasireotide, which targets somatostatin receptors, is currently the only tumor-targeting agent approved for use in patients with Cushing disease. In the phase 3 trial, 26% of patients achieved urinary free cortisol normalization after the 6 months of treatment. However, 73% showed hyperglycemic-related events (14).

An alternative approach to medical therapy for Cushing disease is to more specifically target the corticotroph lineage (15). Corticotroph differentiation occurs following expression of the corticotroph-specific transcription factor Tpit (16); these cells do not express differentiation transcription factors, such as Prop1 and Pit1, that are required for somatotroph, lactotroph, and thyrotroph lineage development (17, 18). Some cell cycle proteins involved in pituitary development are also involved in tumorigenesis (19), specifically serine-threonine cyclin-dependent kinases (CDKs), cyclins, CDK inhibitors, retinoblastoma (Rb) and its complex with transcription factor (Rb/E2F), and pituitary tumor transforming gene (PTTG) (20). However, the contribution of cell cycle proteins to development of lineage-specific tumors has not been well-elucidated.

In this review, we discuss molecular mechanisms underlying the corticotroph lineage-specific cell cycle proteins cyclin E, the CDK inhibitor p27, Rb, and E2F1 in Cushing disease tumorigenesis and consider how cell cycle regulation affects pituitary biology. Then, we present updated data from studies evaluating corticotroph lineage targeting therapy for Cushing disease.

## Overview of cell cycle proteins and pituitary tumorigenesis

Cell division is divided into four phases: S phase (synthesis of DNA), M phase (mitosis), and G1 and G2 (gap) phases. G1 phase occurs before S phase, and G2 precedes M phase. In mammalian cells, this process is driven by CDKs that regulate progression through the phases of the cell cycle (21). Cyclin D (D1, D2, and D3) activates CDK4 and CDK6 and facilitates progression during G1. CDK2/cyclin E (E1 and E2) complexes become active at the end of G1 and participate in the transition from G1 to S phase. At the end of S phase and during G2, cyclin E is substituted by cyclin A (A1, A2) to activate CDK2 and CDK1. Finally, CDK1/cyclin B (mostly B1 and B2) complex is involved in progression through G2 and entry into M phase.

Cell cycle progression is also under the control of negative regulators. Specifically the CDK inhibitors INK4 and Cip/Kip families (22). The INK4 family, including p16, p15, p18, and p19, targets the CDK4/6/cyclin D complexes, while the Cip/Kip family, which consists of p21, p27, and p57, targets the CDK2/cyclin E complex (23).

Finally, the tumor suppressor protein Rb negatively regulates entry into the cell cycle and G1/S progression (24–26) by binding to the transcription factor family E2F to target cell cycle-specific genes for repression, while PTTG, part of the securin family, is associated with cell cycle proteins in G1/S phase and chromosomal instability (27–29).

The Rb heterozygous knockout mouse was the first model linking cell cycle proteins with pituitary tumorigenesis, with nearly 100% developing pituitary tumors by age 12 months (30–32). Several CDK inhibitor knockout models, including p18 and p27, also exhibited pituitary tumors (23, 33), while models of combined CDK inhibitor knockout shortened latency of tumor formation or increased size of pituitary tumors, including knockout of p21/Rb (34), p27/Rb (35), p16/p18 (36), p27/cyclin E (37), p27/p18 (33), p21/p18 (38), and CDK4/p27 (39). Germline, but rarely somatic, mutations (40–42) as well as underexpression or DNA methylation of CDK inhibitors (43) have also been reported in human pituitary tumors.

## Cell cycle proteins and corticotroph lineage

Corticotrophs represent 10–15% of pituitary anterior lobe cells. Corticotroph is the earliest pituitary lineage to initiate cell development and reaches terminal differentiation by expressing lineage-specific transcription factors such as Tpit (16, 44). Cell cycle proteins cyclin E, p27, Rb, and E2F1 exhibit different levels of lineage-specific expression patterns in embryonic cells, adult normal pituitary, and corticotroph adenoma (Table 1).

**Table 1:** Expression of cell cycle proteins in corticotrophs.

	**Expression**			**Corticotroph lineage specificity**	**Evidence of tumorigenesis**	**Cell cycle phase**	**References**
	***Embryo***	***Normal corticotroph***	***Corticotroph adenoma***				
Cyclin E	Precursors +	+	+ (↓)	+++	+	G1/S	(23, 37, 45–47)
p27	Fetal cells +	+	+ (↓)	++	+++	G1/S	(48–52)
Rb	?	+	+ (↓/?)	+/?	+++	G1/S	(31, 53–57)
E2F1	?	+	+ (?)	+++	+/?	G1/S	(58, 59)

↓ *Decreased expression in corticotroph adenoma compared to normal corticotroph*.

### Cyclin E and corticotroph lineage specificity

Cyclin E is upregulated in late G1 and is maintained into S phase, forming a complex with and activating CDK2 at the restriction point shortly prior to entry into S phase. Unique transient expression patterns of cyclin E have been reported in early stages of pituitary cell development. Pituitary progenitor cells in cell cycle progression express cyclin A, D1, D2, and D3, but cyclin E and p57 are only expressed once progenitor cells exit from the cell cycle (23, 46). By the time Tpit is expressed, cyclin E can no longer be detected in corticotroph progenitors (23).

Cyclin E expression is significantly increased in corticotroph adenomas compared to normal pituitary as well as to somatotroph, lactotroph, and non-functional adenomas (45). In a zebrafish model with PTTG-driven corticotroph adenomas, we found cyclin E was significantly upregulated, while cyclin D, p27, and Rb was unchanged (47). By contrast, others have shown that cyclin D is upregulated in non-functioning adenomas and in aggressive non-functioning pituitary adenomas (45, 60, 61).

Transgenic mice overexpressing cyclin E in cells expressing the adrenocorticotrophin (ACTH) precursor proopiomelanocortin (POMC) show abnormal reentry into the cell cycle as well as centrosome instability (37, 62). Molecular analysis suggests that cyclin E levels inversely correlate with expression of the tumor suppressor Brahma-related gene 1 (Brg1), which exerts negative feedback on the glucocorticoid receptor through the *rPomc* promoter (37, 62). Brg1 forms a complex with histone deacetylase 1, the glucocorticoid receptor, and orphan nuclear receptor growth factor 1B; loss of Brg1 disrupts this complex, inducing loss of glucocorticoid negative feedback, as is evident in the clinical phenotype of Cushing disease (62). Indeed, disordered Brg1 and/or cyclin E expression was found in about half of corticotroph tumors derived from 25 patients with Cushing disease, indicating that loss of Brg1 tumor suppression combined with cyclin E upregulation may contribute to corticotroph tumorigenesis (37). Transgenic mice overexpressing cyclin E exhibit pituitary hyperplasia but no pituitary tumors (37), and cyclin E overexpression in a p27 knockout model known to induce pituitary tumor show increased tumor size, further confirming the contribution of cyclin E to corticotroph tumor development and proliferation (37).

Nevertheless, as noted above, about half of 25 Cushing tumors showed an inverse correlation between Brg1 and cyclin E, but the other half did not fit the pattern (37). Also, study of 48 human prolactinoma specimens showed increased expression of both cyclin D1 and cyclin E on immunostaining, and that co-expression correlated with invasiveness (63). These data suggest additional mechanisms of cyclin E regulation in corticotroph lineage specific tumorigenesis.

### p27 and corticotroph lineage specificity

p27, a member of the Cip/Kip family of CDK inhibitors, targets G1/S progression. p27 knockout mice develop enlarging pituitaries by as early as 10 weeks, and tumors in the intermediate lobe show positive POMC expression by 12 months (48–50). Combined Rb/p27 knockout results in even shorter latency of pituitary tumors (35), suggesting two separate pathways for p27 and Rb in G1/S phase deregulation. On immunohistochemistry, p27 labeling is suppressed in corticotroph adenomas and carcinomas (51), and recurrent human pituitary adenomas show lower p27 protein levels than do non-recurrent adenomas (52, 64), supporting the contribution of p27 to corticotroph tumorigenesis. In human specimens, p27 germline mutations are reported as MEN type 4, which manifests as neuroendocrine tumors as well as pituitary tumors, including, but not limited to, Cushing disease (65–67). p27 somatic mutations in human specimens are even rarer (40, 41, 68) and p27 mRNA levels are not altered in corticotroph tumors. Rather, post-translational modifications such as proteolysis or ubiquitination may be involved in downregulating p27 protein levels (69, 70).

### Rb and potential corticotroph lineage specificity

Rb regulation of cell cycle progression was initially studied in *Rb* heterozygous knockout mice as homozygous knockout is lethal (30–32). In humans, individuals who inherit one defective copy of *Rb* have an ~90% risk of developing retinoblastoma at an early age (71). Mice heterozygous for *Rb* do not develop retinoblastoma, but instead exhibit nearly 100% penetrance of pituitary tumors by 12 months (30–32). Pituitary tumors have been reported in the intermediate lobe in *Rb* heterozygous mice (54) and in POMC-specific conditional *Rb* heterozygous mice in which the reporter gene was restricted to the intermediate and anterior lobes (57). Tumors in the former knockout model stained positive for α-melanocyte–stimulating hormone, and neither reported on the presence of tumors in the anterior lobe.

In humans, pituitary neoplasms have not been reported in those with familial retinoblastoma (72). However, loss of an *Rb* allele has been reported in case reports and a few small series of corticotroph adenomas. No allelic loss of *Rb* was seen in a study of 12 human pituitary tumors, including one corticotroph microadenoma and one macroadenoma (53), but among 13 highly invasive human pituitary adenomas or metastatic carcinomas tumors, all showed loss of *Rb*, suggesting a preference for loss of *Rb* in more aggressive corticotroph tumors (55). Indeed, in a patient with adjacent pituitary benign adenoma and carcinoma, Rb expression was significantly reduced in the corticotroph carcinoma but not in the adenoma (56). Given the potential association between aggressive corticotroph tumors and *Rb* loss, as well as the tendency for Rb to complex with the corticotroph-specific E2F1 (59), it is tempting to speculate that Rb-related dysregulation of the cell cycle might have corticotroph preferential pattern, but further investigation is still needed.

### E2F1 and corticotroph lineage specificity

The E2F family of cell cycle proteins, numbered E2F1 through E2F8, includes those that interact with Rb and act on G1 to S phase progression (73). E2F family proteins are expressed ubiquitously, particularly in association with cancer cell cycles and tumorigenesis. Free E2F1 unbound from Rb, the active form of E2F1, binds to target gene promoters and may target cell cycle regulators such as cyclin E and cyclin D (74).

We studied *E2F1* and *POMC* gene regulation in ectopic ACTH-secreting tumors (58). In addition to its cell cycle effects, E2F1 also directly binds to the *POMC* promoter (58). Co-transfection of E2F1 and its heterodimerization partner DP1 enhanced *POMC* promoter activities as well as POMC mRNA levels, while knocking down E2F1 by siRNA-suppressed POMC. E2F1 direct binding and dissociation from POMC promoter region is controlled by site-specific phosphorylation/de-phosphorylation of E2F1 serine 337 (58).

Of note, we found that E2F1 expression is highly specific to corticotrophs in human pituitary tissue (59). By co-staining with human pituitary hormones, we found that E2F1 co-localizes with POMC in normal human corticotrophs, but not with prolactin or growth hormone in lactotrophs or somatotrophs, respectively (59). E2F1 corticotroph specificity also seems to be subclass specific: E2F1 but not E2F3 enhanced POMC promoter activities by deletion mutant *hPOMC* luciferase assays using ectopic ACTH-secreting tumor cells derived from human small cell lung cancer DMS79 cells (58). Others similarly found that overexpression of E2F3 is not sufficient to produce pituitary tumors, even though it leads to pituitary hyperplasia (75).

Whether E2F1 is involved in pituitary development or tumorigenesis is unknown. Double *Rb* and *E2F1* gene knockout mice show fewer pituitary tumors than do *Rb* knockout mice with intact E2F1 (76), suggesting a role of E2F1 in pituitary tumorigenesis, but *Rb* heterozygous mice with double E2F4 knockout also show increased tumor incidence (77).

### E2F1 as part of downstream signaling of EGFR

Epidermal growth factor receptor (EGFR) is expressed to varying degrees in human pituitary tissue, including corticotroph adenomas (78), and EGFR regulates POMC transcription and ACTH production (79). However, mechanisms for corticotroph-specific tumor induction by EGFR or for EGFR upregulation of ACTH/POMC expression have not been clearly elucidated.

*rPomc* promoter-driven EGFR transgenic mice show pituitary corticotroph tumors in the anterior and intermediate lobes and demonstrate phenotypes similar to those in human Cushing disease, including obesity, glucose intolerance, and adrenal hyperplasia (59). E2F1 and phosphorylated serine 337-E2F1 were both upregulated in these tumors, but were attenuated with EGFR inhibition. Although EGFR is expressed in other lineages of aggressive pituitary tumors (80), our findings suggest that EGFR signaling induces E2F1-mediated POMC transcription and corticotroph adenoma pathogenesis. This pathway may therefore be a candidate for corticotroph-specific targeted therapy in patients with Cushing disease.

Recently, gain-of-function somatic mutations in *USP8* were reported in 30–50% of Cushing tumors (81, 82). USP8 is a deubiquitinase that protects EGFR from lysosomal degradation. These mutations lead to a higher rate of USP8-induced EGFR deubiquitination, increasing EGFR pathway stimulation, and ultimately increasing ACTH secretion (81, 82). However, the mechanisms and influence of *USP8* mutations on Cushing tumors are not fully understood. EGFR expression is unchanged comparing mutated vs. non-mutated tumors (83), and phenotypic features of aggressive tumors, particularly larger tumor size, are not consistently seen (81, 83, 84). Further study of these mechanisms and the clinical implications of *USP8*-mutated Cushing tumors is ongoing.

### G1/S phase is the key in corticotroph tumors

The cyclin E promoter has binding sites for E2F1, which, in turn, upregulates cyclin E mRNA (74, 85). E2F1 also binds Rb, while p27 targets the CDK2/cyclin E complex. This suggests cyclin E, p27, E2F1, and Rb could interact to regulate cell cycle progression through G1 (74).

**Figure 1:**
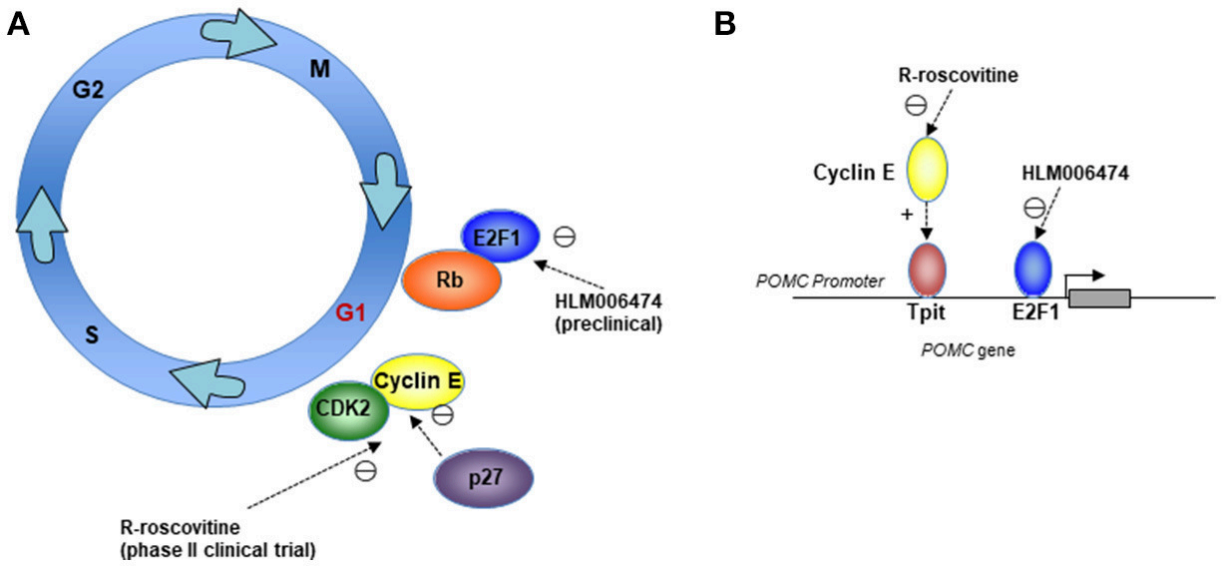
Mechanisms for therapeutically targeting the cell cycle G1/S phase in corticotroph adenomas. Cartoons depicting **(A)** Cell-cycle targets for the CDK2/cyclin E inhibitor R-roscovitine, which is being investigated in a phase II clinical trial, and the E2F inhibitor HLM006474, which is being investigated in preclinical models; and **(B)** Suppression of the POMC promoter through cyclin E and E2F1 by R-roscovitine and HLM006474.

Dysregulation of G1/S transition is often seen in human cancers. Our data suggest that lineage-specific amplification of cyclin E/E2F1 signals contribute to uncontrolled POMC transcription and autonomous ACTH production in corticotroph tumors (86), and cyclin E, p27, Rb, and E2F1 have also been shown to affect G1/S transition in corticotrophs (23, 58, 59, 62). Targeting G1/S could therefore be a reasonable therapeutic approach in patients with corticotroph tumors (Figure 1).

## Therapeutic targeting of cell cycle proteins in corticotroph tumors

Several small molecule CDK inhibitors are being evaluated in different pituitary tumor types (87, 88). To date, such agents have proven effective in preclinical studies (39, 47, 86). The first study treated CDK4/p27 double knockout mice harboring anterior pituitary tumors with flavopiridol, an inhibitor of CDK1, CDK2, CDK4, CDK6, and CDK7 that causes cell-cycle arrest at G1 and G2, and showed shrinkage of pituitary tumor size as well as prolonged survival (39). Using the small molecule E2F inhibitor HLM006474, originally developed as a therapy for melanoma (89, 90), we showed dose-dependent suppression of POMC mRNA expression in primary cultures of surgically resected Cushing tumor tissue, but no suppression of growth hormone used as a control (59). Despite its lack of specificity for E2F1, the corticotroph-specific effects suggest this agent may be useful in POMC-producing tumors.

Using a germline transgenic zebrafish model overexpressing PTTG in pituitary corticotrophs to recapitulate human Cushing disease, we tested several small molecule CDK inhibitors on corticotroph adenomas *in vivo*, including flavopiridol, R-roscovitine (seliciclib; primarily an inhibitor of CDK1 and CDK2 in late G1 but not an inhibitor of CDK4 and CDK6), olomoucine (CDK1, CDK2), PD-0332991 (CDK4/6 in early G1), and CAY10572 (CDK7 in S phase). Addition of PD-0332991 or CAY10572 to the culture medium of double transgenic embryos generated by breeding the POMC:PTTG model with transgenic zebrafish expressing green fluorescent protein (eGFP) resulted in no significant change in pituitary expression of POMC-eGFP compared with controls, while a modest reduction of ~20% was observed in the olomoucine-treated group (47). By contrast, R-roscovitine-treated embryos exhibited ~40% reduction in pituitary POMC-eGFP expression compared with controls (*P* < 0.02) (47).

R-roscovitine is a second-generation CDK inhibitor that interrupts ATP binding of CDK. It has a relatively broad range of activity, but primarily targets the CDK2/cyclin E complex (91). R-roscovitine has been studied in patients with nasopharyngeal cancer, non-small lung cancer, and B-cell malignancies, but few clinical trial data have been reported (92). Side effects include mild to moderate fatigue, nausea/vomiting, constipation, fever, cough, and elevated liver enzymes, which typically resolved after drug discontinuation (93).

In AtT20 cells, a murine corticotroph adenoma cell line commonly used as a model for Cushing disease, as well as in *PTTG* zebrafish models, R-roscovitine treatment significantly suppressed POMC expression both *in vitro* and *in vivo* (47). Plasma ACTH, corticosterone levels, and tumor size were significantly reduced in AtT20 cells xenografted in mice with R-roscovitine treatment (47), and POMC mRNA and ACTH levels were dose dependently suppressed in primary cultures derived from human corticotroph adenomas (86). In addition to its cell cycle effects, R-roscovitine also has direct inhibitory effects on the POMC promoter (58, 86). Deletion mutant and point mutant *rPomc* luciferase assays showed that R-roscovitine suppressed the *rPomc* promoter by targeting the Tpit binding region (TCACACC) and suppressed protein expression of cyclin E and E2F1 in a dose-dependent manner (86), suggesting that suppression of Tpit expression is mediated by cyclin E/E2F1 reciprocal regulation. Importantly, viable tumor cell numbers were largely unchanged despite decreased ACTH concentration in the culture medium of primary cultures (86). In ectopic ACTH-secreting tumor xenografted mice, R-roscovitine similarly suppressed POMC/ACTH secretion, but it did not alter tumor proliferation in DMS79 cells (58, 94). The inhibitory effect of this agent in human corticotroph tumors preferentially targets ACTH expression rather than tumor cell growth suggests other corticotroph mechanisms independent of cell cycle regulation may be present. A phase II study of R-roscovitine (seliciclib) is currently underway for the treatment of patients with *de novo*, recurrent, or persistent Cushing disease (ClinicalTrials.gov NCT02160730).

## Conclusions

Corticotrophs are sensitive to changes in cell cycle regulation, and evidence suggests involvement of corticotroph lineage-specific cell cycle regulators such as cyclin E, p27, Rb, and E2F1 in tumorigenesis. Currently, only R-roscovitine, a cyclin E/E2F1 inhibitor, is in clinical development, but it is likely that other agents targeting these factors will prove attractive as novel medical therapy options for patients with Cushing disease.

## Author contributions

TA and N-AL conceived and designed the project. TA collected and analyzed the evidence and prepared the draft manuscript. TA and N-AL contributed to manuscript revisions and approved the submitted version.

### Conflict of interest statement

The authors declare that the research was conducted in the absence of any commercial or financial relationships that could be construed as a potential conflict of interest.
